# Balloon-Occluded Hepatic Radioembolization with Yttrium-90 (b-TARE) in Selected Patients with Unresectable Heterogeneous Hepatocellular Carcinoma (HCC): A Safe and Effective Approach to Improve the Dose Rate

**DOI:** 10.3390/diagnostics15243237

**Published:** 2025-12-18

**Authors:** Leonardo Teodoli, Nicolò Ubaldi, Claudio Trobiani, Federico Cappelli, Sara Ungania, Pierleone Lucatelli, Rosa Sciuto, Giulio Vallati

**Affiliations:** 1 Department of Radiology and Diagnostic Imaging, Interventional Unit, IRCCS Regina Elena National Cancer Institute, 00144 Rome, Italy; 2 Interventional Radiology Unit, Department of Medical Surgical Sciences and Translational Medicine, Sapienza—University of Rome, Sant’Andrea Hospital, 00189 Rome, Italy; 3 Physics Department, “IRCCS Istituto Nazionale Tumori Regina Elena”, 00138 Rome, Italy; 4 Interventional Radiology Unit, Department of Diagnostic Medicine and Radiology, UOC Radiology, Azienda Ospedaliera Universitaria Policlinico Umberto 1, 00161 Rome, Italy; pierleone.lucatelli@gmail.com

**Keywords:** hepatic radioembolization, balloon microcatheter, SPECT-CT, b-TARE

## Abstract

**Background/Objectives:** To evaluate the efficacy of balloon occlusion trans-arterial hepatic radioembolization with Yttrium-90 (b-TARE) in optimizing dose activity in patients with large or multifocal Hepatocellular Carcinoma (HCC) lesions with heterogeneous macroaggregate distribution by retrospectively comparing outcomes with a similar cohort treated with standard TARE. **Methods:** This single-center restrospective study included sixty-three consecutive patients with unresectable HCC treated with TARE, of whom 24/63 had balloon-occluded TARE and 39/63 had standard TARE. Both cohorts included large or multifocal HCC lesions characterized by heterogeneous macroaggregate distribution, also in relation to the angiosome framework. The impact of b-TARE was analyzed using 2D and 3D dosimetry with dedicated software on post-procedural SPECT-CT. Dosimetric b-TARE results were retrospectively compared with standard TARE. **Results:** Both 2D and 3D SPECT-CT analyses demonstrated a better dosimetry profile in the b-TARE group. Concerning 2D evaluation, the activity intensity peak was significantly higher in the b-TARE group compared to the TARE group (998.6 ± 394.9 vs. 578.8 ± 313.3, *p* = 0.004). Regarding 3D dose analysis, the mean intra-lesion dose administered was significantly higher in the b-TARE group (162.7 ± 54.3 Gy vs. 111.2 ± 44.5 Gy, *p* = 0.01). There was no increase in significant complications or in the mean dose delivered to the normal liver in the b-TARE group. **Conclusions:** The employment of balloon occlusion in TARE was associated with a higher activity intensity peak and lesion absorbed dose on voxel-based dosimetry, compared to standard TARE, in patients with heterogeneous HCC and uneven macroaggregate distribution, without increasing mean non-tumoral liver dose.

## 1. Introduction

Endovascular trans-arterial treatments represent the standard of care for intermediate HCC that is not amenable to curative treatments, and they are also an alternative in early-stage disease if surgery or ablation is contraindicated [1,2,3].

The temporary occlusion of a pathologic artery feeder can act as a game changer in liver embolization, introducing a pressure gradient-driven approach to microsphere delivery [4,5,6]. Using a balloon microcatheter to transiently occlude the primary tumor-feeding artery, the technique exploits the redistribution of blood flow through collateral interlobar and intersegmental channels. This hemodynamic shift creates a favorable pressure gradient that selectively redirects embolic material toward low-resistance tumoral neoangiogenic territories, thereby enhancing tumor targeting and microsphere deposition [7]. This effect was confirmed both in ex vivo and in vivo models, depicting a better embolization profile [8,9].

To date, scientific efforts have been pursued to understand the safety profile and the therapeutic efficacy of balloon-occluded trans-arterial chemoembolization [10,11]. Several in vivo methodologies have been described to assess catheter-based embolization treatments. These include the non-enhanced cone-beam CT (CBCT) performed immediately after the procedure to visualize microparticle deposition—as the embolic agents are mixed with contrast media—and to evaluate signal-to-noise and contrast-to-noise ratios based on pseudo-attenuation measurements of the target nodule and surrounding liver parenchyma. Alternatively, histopathological analysis of explanted liver specimens has been performed to directly confirm and characterize microparticle distribution within the treated tissue [9,12,13]. However, these represent surrogate markers and are generally not reliable for balloon trans-arterial radioembolization (b-TARE) due to the limited number of transplanted cases available for histological assessment and the reduced mixing of microspheres, which diminishes the effectiveness of CBCT [11,14,15].

In this context, the angiosome framework offers a physiologically grounded approach to evaluating intrahepatic microsphere distribution according to arterial perfusion territories. It enables the assessment of radiopharmaceutical distribution in TARE by linking 99mTc-MAA uptake to specific hepatic arterial distribution areas. This approach refines regional dosimetry by correlating tracer deposition with the actual perfused parenchyma [16]. Cases where the 99m-Tc-MAA deposition in the simulation phase is heterogeneous—commonly occurring in large or multifocal tumors, necrotic lesions, or multiple or extrahepatic feeding arteries—reflect variation in the hepatic arterial perfused volumes or angiosomes [17] and can undermine the predictive reliability of pretreatment dosimetry, thereby complicating optimal activity prescription [18,19]. A possible remedy may lie in selective b-TARE, which helps redirect blood flow towards the tumor, reducing perfusion of competing segments, minimizing reflux, and thus promoting more uniform intra-lesional microsphere deposition [4,5,6,7,8,9]. A SPECT-CT scan performed after TARE is a reliable technique for measuring the in vivo efficacy of the procedure, as it enables accurate dosimetric evaluation through dedicated radiological and nuclear medicine software. Hence, a detailed analysis of activity distribution and calculation of effective absorbed dose in Gray (Gy) can be performed, which adds data to the limited dosimetry-based evidence currently available for b-TARE.

This study aims to evaluate the role of the b-TARE by assessing its impact on the distribution of administered activity between the heterogeneous lesion and the healthy liver through objective absorbed dose assessment and by correlating these data with both the efficacy and safety of the procedure.

## 2. Materials and Methods

This study was approved by the Ethics Committee of the “Istituto Regina Elena Istituto di Ricovero e Cura a Carattere Scientifico,” with the protocol number code CE RS1561/21(2547), and the approval date was the 16 December 2021. Informed consent was obtained from all individual participants enrolled in the study.

All procedures were performed at the Interventional Radiology angiosuite within the Radiology Department of Regina Elena National Tumor Institute—IFO, IRCCS in Rome. Our interventional radiology team has extensive experience in this field, performing an average of 100 TARE procedures annually.

HCC diagnosis in at-risk patients followed guideline-concordant imaging pathways and LI-RADS (v2018) categorization [20].

The indications for TARE were given by a multidisciplinary board composed of a transplant surgeon, an interventional radiologist, an oncologist, a radiotherapist, and a hepatologist.

The rationale behind b-TARE is to improve the distribution of the macroaggregate in selected cases, where the heterogeneous macroaggregate distribution could otherwise negatively affect dose delivery during treatment, a process that can be better understood and guided through the angiosome framework. Therefore, in this study, all consecutive eligible patients treated during the defined time window underwent b-TARE, and outcomes were then retrospectively compared with a similar cohort with heterogeneous macroaggregate distribution treated with standard TARE. The use of the balloon was independent of lesion size or extension.

Inclusion criteria were naïve patients with Child–Pugh scores up to B8 and Barcelona Clinic Liver Cancer (BCLC) stages up to B, who were not eligible for curative treatments. Exclusion criteria were extrahepatic disease, high-flow portal-arterial fistula, platelet count  <  50,000, and bilirubin level  > 3 mg/dL. All patients underwent physical examination, laboratory tests, and diagnostic imaging studies within 30 days of treatment.

### 2.1. b-TARE Technique and Outcome Measurements

All procedures were performed by two interventional radiologists with more than 15 years of experience in TARE each.

The procedure protocol was standardized for both standard and balloon-occluded TARE.

All procedures were performed through US-guided common femoral artery access under local anesthesia. In all cases, a treatment simulation by injection of 99m-Tc-MAA was performed. Liver tumor vascularization map and lesion feeder detection were obtained with digital subtraction angiography (DSA) in anterior–posterior and right-anterior oblique 25° projections, performed with a 5 French diagnostic catheter in the common hepatic artery.

Arteries that could have contributed to non-target radioembolization were embolized with coils. When the correct position for embolization was obtained, injection of 99m-Tc-MAA was performed employing a standard 2.7 Fr microcatheter (Occlusafe, Terumo Europe NV, Leuven, Belgium).

Between 1 and 20 h after the simulation procedure, a 99m-Tc-MAA SPECT-CT scan (Symbia IntevoTM system; Siemens, Erlangen, Germany) [21] was performed to verify the correct distribution of Tc-MMA in HCC nodules, to exclude any extrahepatic shunts, to quantify the lung shunting fraction (LSF), and to calculate the dose of Yttrium—90 (90Y) for the treatment phase. The treatment was performed 7 ± 2 days after the simulation procedure.

The optimal location for the balloon microcatheter in b-TARE was chosen based on the information obtained by angiographic images, CBCT, and subsequent SPECT-CT scans.

The balloon microcatheter employed was a 2.8 Fr microcatheter (1.9 Fr at the tip) with a 10 mm length balloon on the tip (Occlusafe, Terumo Europe NV, Leuven, Belgium) [22]; all procedures were performed using a hydrophilic guidewire (GT guidewire, Terumo Europe NV, Leuven, Belgium).

Once the balloon microcatheter was positioned within the target vascular segment, the arterial pressure at the tip of the microcatheter was measured using an invasive arterial pressure measurement kit. Thereafter, the balloon was inflated with a solution 1:4 of contrast media/saline under fluoroscopic control in order to obtain a drop in the balloon-occluded arterial stump pressure (BOASP) [22,23,24].

For standard TARE, a 2.7 F standard microcatheter (Progreat; Terumo Europe NV, Leuven, Belgium) was used.

TARE was then performed with 90Y resin microspheres (SIR-Spheres^®^, Sirtex SIR-Spheres Pty Ltd., Woburn, MA, USA) in TARE and b-TARE [25]. The amount and dose of 90Y-resin microspheres administered were defined by the Radiotherapy unit based on SPECT-CT scans acquired after simulation with 99m-Tc–MMA [26].

Post-treatment 90Y SPECT-CT (Symbia IntevoTM system; Siemens, Erlangen, Germany) scans were performed between 1 and 20 h after radioembolization [26] to evaluate the 90Y-microspheres distribution and to calculate 2D and 3D dosimetry using a dedicated fused image software for medical image analysis specific for radiotherapy (MIM Maestro^®^ ver. 6.1.7 by MIM Software Inc., Science Park Drive-Suite 180 Cleveland), as described below.

### 2.2. Study Outcome

Technical success was defined as a composite outcome measurement: ability to place the balloon microcatheter inside the targeted vascular segment, a drop in BOASP, and a qualitative assessment of 90Y microspheres in the target tumor [7,22,27].

Safety of b-TARE was also evaluated in terms of adverse events (AEs) incidence and evaluation of liver function tests and routine laboratory tests before and after the procedure (within 36 h). The AEs were categorized according to the CIRSE classification system [28]. The laboratory test modifications, pre- and post-procedure, were evaluated according to the Common Terminology Criteria for AE v5.0 [29]. Post-embolic syndrome was defined as the onset of post-procedure fever and/or nausea and/or pain (pain score  >  6) on a visual analog scale and was evaluated prior to discharge. Liver function tests included bilirubin, transaminases, alkaline phosphatase, γ-GT, and albumin. The routine laboratory examinations included a full blood count and the coagulation profile.

Efficacy of b-TARE was evaluated by using a multilevel dosimetric approach.

Firstly, the accuracy and intensity of 90Y-microspheres activity distribution were evaluated by comparing the 2D activity intensity peak (Pixel Value) of the signal along a line crossing the treated area in both groups expressed in grayscale. The higher the peak, the more intense the signal inside the treated area.

Then, the 3D effective dose (Gy) absorbed by target areas and normal liver parenchyma, per unit cumulated activity (GBq), was calculated based on the activity distribution on SPECT-CT scans using a certified kernel convolution method for activity-to-dose calculation. Lesion and normal liver parenchyma volumes were delineated on the MIM 6.1.7 workstation (MIM Software Inc., Cleveland, OH, USA), and dose calculation was performed on these volumes [30,31]. For each patient, the mean absorbed dose (<D>) in Gy from normal liver parenchyma and tumors was compared with guideline-based thresholds according to the radiotherapy guidelines. Endpoints were a <D> > 100 Gy in the tumors to reach radio-necrosis and a <D> < 40 Gy in the whole liver parenchyma to prevent radiation damage and liver failure [32].

Calculating the tumor-to-non-tumor ratios both on 99mTc-MAA SPECT and on post-treatment 90Y imaging for each patient could be useful in the future to serve as their own control; however, this comparison has the intrinsic limitation that 99mTc-MAA and 90Y microspheres are not identical particles, and their intrahepatic distributions may differ.

### 2.3. Statistical Analysis

Continuous variable group normality was tested using the Kolmogorov–Smirnov Z test. Continuous data were described as the mean value ± SD, whereas non-Gaussian data were described with the median and confidence interval of 95%. Student’s t-test or the Mann–Whitney test was used according to the data distribution. A *p*-value of less than 0.05 was defined as statistical significance. Each *p*-value was calculated using a two-tailed significance level. Statistical analysis was performed with MedCalc 15.0 software (MedCalc, Mariakerke, Belgium).

## 3. Results

From January 2022 to May 2024, 63 patients (mean age 67.1  ±  14.0 years [range, 41–86 years]; 40 males, 23 females) with 240 HCC nodules (average 3.8 HCC nodules/patient) were consecutively enrolled. In total, 39/63 (61.90%) patients with 156 HCCs underwent standard TARE, while 24/63 (38.09%) patients with 84 HCC nodules underwent b-TARE. Clinical and demographic characteristics of the cohort are shown in Table 1.

No differences were found between the two groups in terms of liver function parameters or disease burden; all patients enrolled were grade B in the BCLC grading system; the median number of nodules treated per patient was 3.5 [CI 95% 1–9.2] in the b-TARE group and 4 [CI 95% 3–5.65] in the TARE group (*p* = 0.31); the median nodule diameter was 42 mm [CI 95% 29.9–75.6] in b-TARE group and 34 mm [CI 95% 31.0–47.6] in TARE group (*p* = 0.43); the nodules treated were segmental and sectional with no specific distribution between the two groups. Also, no differences were found between the two groups in terms of dose activity administered: mean activity administered in GBequerel (GBq) was 1.74 [CI 95% 1.60–2.02] in the TARE group and 1.67 [CI 95% 1.62–1.88] in the b-TARE group. LSF was similar in the two groups (7% in the TARE group and 6% in the b-TARE group) (Table 2).

The technical success of b-TARE was 96%. In one patient, the BOASP reduction in pressure was not achieved.

No post-procedural complications were reported in either group.

Regarding efficacy, b-TARE demonstrated a better dosimetrical profile both in 2D and 3D analysis. Concerning 2D evaluation, the activity intensity peak (calculated in grayscale) was significantly higher in the b-TARE group compared with the TARE group (998.6 ± 394.9 vs. 578.8 ± 313.3, *p* = 0.004), demonstrating that in b-TARE, a higher number of 90Y-microspheres were delivered to the lesion using the same administered dose. Regarding 3D dose analysis (expression of absorbed dose in Gy), the mean dose <D> absorbed to the treated lesions was significantly higher in the b-TARE group than in the TARE group (162.7 ± 54.3 Gy vs. 111.2 ± 44.5 Gy, *p =* 0.01). With no significant increase in the mean dose delivered to the normal liver (29.5 ± 5.8 Gy vs. 29.0 ± 8.9 Gy, *p* = 0.70), Table 3.

## 4. Discussion

In this retrospective analysis, b-TARE demonstrated a superior and more clearly delineated dose distribution compared with conventional TARE in similar patient cohorts with both heterogeneous HCC lesions and macroaggregate distribution. These technical advantages could lead to several clinical benefits in different fields of application.

The angiosome model enhances the physiological interpretation of microsphere distribution in TARE, allowing regional dosimetry to reflect the actual perfused parenchyma. When 99mTc-MAA uptake is uneven across hepatic arterial angiosomes—as often seen in large, multifocal, or necrotic tumors, or with multiple arterial feeders—pre-treatment dosimetry becomes less predictive, complicating activity optimization [16,17,18,19]. In such scenarios, selective b-TARE with intra-procedural flow modulation may improve dose conformity.

**Figure 1:**
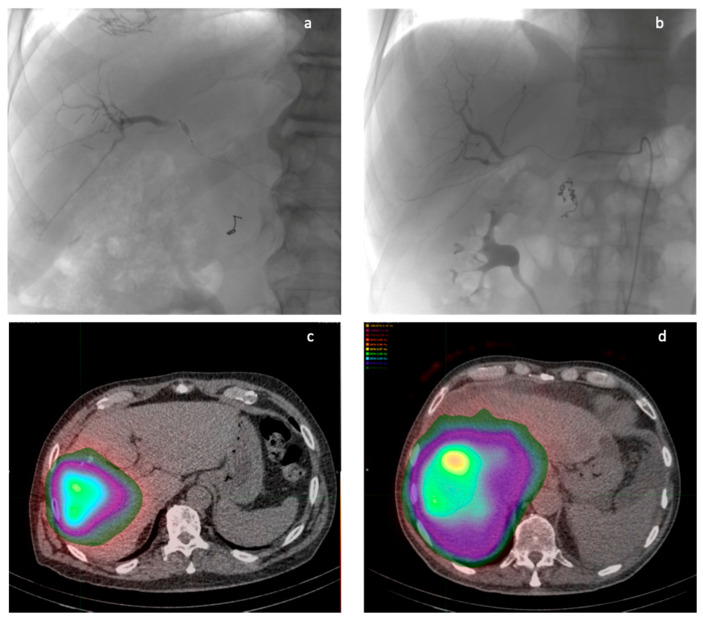
Comparison of two similar cases in terms of tumor burden, activity administered (1.88 GBq b-TARE, 1.90 GBq TARE), and position of the microcatheter’s tip before injection of 90Y microspheres. (**a**,**b**) show similar microcatheter tips in digital angiography, respectively, in the b-TARE patient and standard TARE patient; (**c**,**d**) show 90Y SPECT-CT fused image dose distribution, respectively, in the b-TARE patient and standard TARE patient.

**Figure 2:**
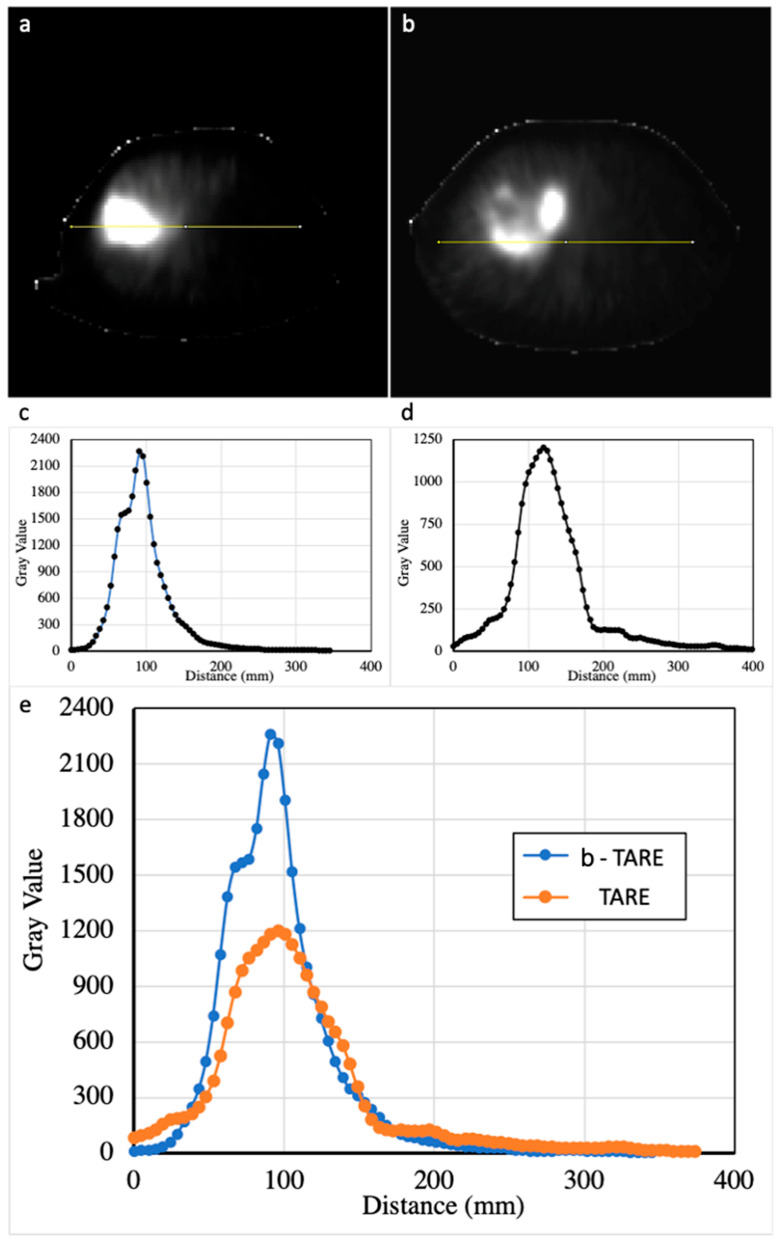
Comparison between b-TARE (**a**,**c**) and standard TARE (**b**,**d**) in post-treatment 90Y SPECT images in patients with similar anatomy, lesion size and location; (**a**,**b**) 2D profiles generated at a central slice on activity distribution SPECT images, (**c**,**d**) activity intensity peak in gray-scale per mm along the profile crossing the lesion; (**e**) comparison of the 2D activity intensity peaks.

The balloon microcatheter allowed for a more accurate dose distribution in this population, with the same amount of administered activity. Figure 1 compares two cases with similar tumor burden, illustrating the different dose distributions resulting from analogous activity administered from comparable arterial branches. A higher signal intensity, represented by a higher 2D peak, was demonstrated in the b-TARE group. This result can be interpreted as a maximization of the 90Y-microspheres uptake and deposition inside the lesion compared to the TARE group. Moreover, 3D dosimetry demonstrated that a balloon-occluded microcatheter permits achieving a higher mean absorbed dose (>150 Gy) in the lesion, well above the threshold (100 Gy), allowing for radio-necrosis without increasing the mean absorbed dose to the surrounding healthy liver parenchyma. This could result in an improvement in the efficacy and safety profile in radioembolization procedures, with a higher therapeutic dose distributed to the target lesions (Figure 2).

In radioembolization procedures, SPECT-CT represents the standard of care for post-procedural evaluation [30,31], allowing dose distribution assessment and personalized dosimetry of 90Y-microspheres. The absorbed dose (Gy) within the lesion and in the normal liver parenchyma, based on SPECT-CT scans, is essential data for coupling it to the radiobiological effect and allows for predicting the treatment success and/or complications such as radioembolization-induced liver disease [33].

Based on our results, we suggest that b-TARE may play a significant role even in patients with large or multifocal lesions, necrotic with multiple or extrahepatic feeding arteries, and inhomogeneous macroaggregate distribution. In this context, b-TARE could serve as an alternative to combined procedures, offering potential advantages, such as a less complex, single-phase technique and the additional benefit of nuclear medicine imaging for evaluating therapeutic efficacy. In terms of patient convenience and cost, combined locoregional therapies often require overnight hospitalization, whereas b-TARE can typically be performed as an outpatient procedure. The possibility of performing both simulation and treatment for 90Y therapy on the same day has been shown to be feasible, opening the opportunity for a single-day outpatient procedure [34,35]. Complications are rare in both radioembolization and combined treatments; however, more serious potential complications are associated with combined treatments in cases of lesions near vital anatomical structures [34].

B-TARE can also be a valid technique in radiation segmentectomy, indicated in patients with lesions limited to ≤2 liver segments, with the intent to administer higher absorbed doses to the perfused target volume without overdosing non-tumoral tissue [36].

In some patients with unilobar disease, the future liver remnant is insufficient to permit resection. Unilobar treatment allows for the delivery of higher absorbed doses, as part of the liver remains untreated, thereby enhancing tumor control. Additionally, it can induce contralateral lobar hypertrophy, potentially enabling bridging previously ineligible patients to curative resection. Radiation lobectomy is a valuable strategy in downstaging/bridge-to-transplant settings, incorporating a biologic test of time that may help identify patients most likely to benefit from resection [32].

Thanks to the selective irradiation of the lesion, saving as much healthy parenchyma as possible, b-TARE may also entail a viable treatment strategy for patients with worse liver function. B-TARE could be exploited in those patients who already had locoregional treatments or surgical resection, in whom maximizing dose concentration within the target while minimizing non-tumoral liver exposure may be particularly valuable [32,36,37,38].

In view of the different caliber of the balloon microcatheter compared to the standard microcatheter commonly used in radioembolizations, considering the different internal lumens of the microcatheters (1.9 Fr vs. 2.7 Fr), it would be interesting to verify its performance, in terms of activity distribution, with different carrying microparticles, specifically glass microparticles (22 ± 10 mm) and biodegradable poly L-lactic acid microparticles (30 ± 10 mm) (smaller average size compared with resin microparticles (32 ± 10 mm) [39].

## 5. Limitations

Limitations of this study are its retrospective nature and the limited cohort of patients who underwent the radioembolization procedure. A matched group of patients based on tumor stage, load, location, and distribution, comorbidities, and biology of cancer, prospectively, would be ideal for the correct comparison.

Imaging modality limitation: Bremsstrahlung SPECT/CT was used for post-therapy imaging during the study window. Contemporary guidance recommends 90Y-PET/CT when available for improved quantification.

There is a high risk of residual confounding of patients eligible for b-TARE, which can be overcome with robust comparative data; preferably, a randomized controlled study is needed.

A further limitation is that we did not systematically verify complete tumor perfusion after balloon occlusion with DSA and cone-beam CT, which is highly recommended to ensure adequate lesion coverage and also to verify the efficacy of balloon inflation.

Finally, we acknowledge that we did not perform a cost-effectiveness analysis, and while the balloon microcatheter adds device cost and procedural time, future prospective studies are needed to determine whether these are justified by the potential dosimetric and procedural advantages observed in complex, selected cases.

## 6. Conclusions

This retrospective analysis demonstrates that balloon-occluded TARE provides a more favorable dosimetric profile compared with conventional TARE in similar cohorts of patients with both heterogeneous HCC and macroaggregate distribution, achieving higher absorbed doses within the tumor without increasing exposure to healthy parenchyma. The angiosome framework enables a physiological interpretation of heterogeneous 99mTc-MAA distribution, highlighting cases where selective b-TARE with intra-procedural flow modulation can optimize microsphere delivery and improve dose conformity. These findings suggest that b-TARE may broaden therapeutic opportunities for patients with unresectable HCC (Stage B—BCLC), including those with large or unilobar disease, offering advantages in settings such as heterogeneous or unreliable 99mTc-MAA distribution, radiation segmentectomy requiring high intratumoral dose intensity, or patients with minimal hepatic reserve, where enhanced dose conformity and sparing of non-tumoral parenchyma may be most impactful.

## Figures and Tables

**Table 1:** Demographic characteristics.

	TARE	b-TARE	*p*
Patient number	39	24	
Sex (M/F)	31/8	21/3	0.8
Age, year (mean value ± SD)	69.7 ± 11.8	66.8 ± 14.9	0.28
Nodule number	156	84	
Nodule/patient (mean value)	4	3.5	0.31
Nodule diameter (mean value)	34	42	0.43
Child—Pugh *N* (%)			0.9
A5	18 (46.16%)	11 (45.83%)	
A6	7 (17.95%)	5 (20.84%)	
B7	12 (30.77%)	6 (25%)	
B8	2 (5.12%)	2 (8.33%)	
Etiology *N* (%)			0.81
Alcohol related cirrhosis	6 (15.38%)	5 (20.83%)	
HBV	7 (17.95%)	7 (29.17%)	
HCV	22 (56.41%)	11 (45.83%)	
NASH	4 (10.26%)	1 (4.17%)	
MELD (mean value ± SD)	9.8 ± 1.9	10.1 ± 1.4	0.71
Indication			0.6
Downstaging	3 (7.69%)	3 (12.5%)	
Bridging	8 (20.51%)	7 (29.16%)	
Palliative	28 (71.80%)	14 (58.34%)	

TARE—trans-arterial radioembolization; b-TARE—balloon-occluded trans-arterial radioembolization; SD—standard deviation; HCV—Hepatitis C virus; HBV—Hepatitis B virus; NASH—non-alcoholic steatohepatitis; MELD—model for end-stage liver disease.

**Table 2:** Procedure features.

	TARE	b-TARE	*p*
Nodule features			
N nodule	156	84	
Nodule diameter (mean value)	34	42	0.43
Dimension max diameter. mm. (median CI 95%)	40.4 (38.3–42.2)	46.1 (42.4–48.0)	0.32
Range maximum diameter (min–max)	5–87	9–84	
Activity GBq (mean value)	1.74	1.67	0.24
Activity max GBq	2.7	2.05	
LSF	7%	6%	0.53

TARE—trans-arterial radioembolization with standard technique; b-TARE—balloon occluded trans-arterial radioembolization; CI—confidence interval; GBq—giga bequerel; LSF—lung shunting fraction.

**Table 3:** Comparison of the activity intensity and absorbed dose in TARE and b-TARE.

90Y SPECT	TARE	b-TARE	*p*
2D evaluation Activity intensity peak (gray value)	578.8 ± 313.3	998.6 ± 394.9	0.004
3D evaluation <D> (Gy)	111.2 ± 44.5	162.7 ± 54.3	0.01
<D> in whole liver parenchyma (Gy)	29.0 ± 8.9	29.5 ± 5.8	0.70

90Y: Yttrium-90, SPECT: Single Photon Emission Computed Tomography; <D>: mean absorbed dose.

## Data Availability

The data presented in this study are available on request from the corresponding author due to privacy concerns.
